# Supplementary material for: Intracellular Hydrolysis of Small-Molecule O-Linked N-Acetylglucosamine Transferase Inhibitors Differs among Cells and Is Not Required for Its Inhibition
Source: Molecules. 2020 Jul 25;25(15):3381. doi: 10.3390/molecules25153381 (PMC7436030; doi:10.3390/molecules25153381)
Supplement: Supplementary file 1 [file molecules-25-03381-s001.pdf]

Intracellular Hydrolysis of Small-Molecule *O*-Linked *N*-Acetylglucosamine Transferase Inhibitors Differs among Cells and Is Not Required for Its Inhibition

Elena M. Loi [1,2], Matjaž Weiss [1], Stane Pajk [1], Martina Gobec [1], Tihomir Tomašič [1], Roland J. Pieters [2] and Marko Anderluh [1,*]

[1] Chair of Pharmaceutical Chemistry, Faculty of Pharmacy, University of Ljubljana, 1000 Ljubljana, Slovenia; Elena.Maria.Loi@ffa.uni-lj.si (E.M.L.); Matjaz.Weiss@ffa.uni-lj.si (M.W.); Stane.Pajk@ffa.uni-lj.si (S.P.); Martina.Gobec@ffa.uni-lj.si (M.G.); Tihomir.Tomasic@ffa.uni-lj.si (T.T.)

[2] Department of Chemical Biology & Drug Discovery, Utrecht Institute for Pharmaceutical Sciences, Utrecht University, P.O. Box 80082, NL-3508 TB Utrecht, The Netherlands; R.J.Pieters@uu.nl (R.J.P)

* Correspondence: Marko.Anderluh@ffa.uni-lj.si; Tel.: +386-1-4769-639

Supporting information

Table of Contents

Organic synthesis of OSMI-4b and derivatives OSMI-4a and OSMI-4 DKP **S1**

Cellular permeability (LC-MS chromatograms) **S2-7**

IC_{50} curves measured with fluorescent activity assay **S8**

IC_{50} curves measured with UDP-Glo Assay **S9**

Organic synthesis

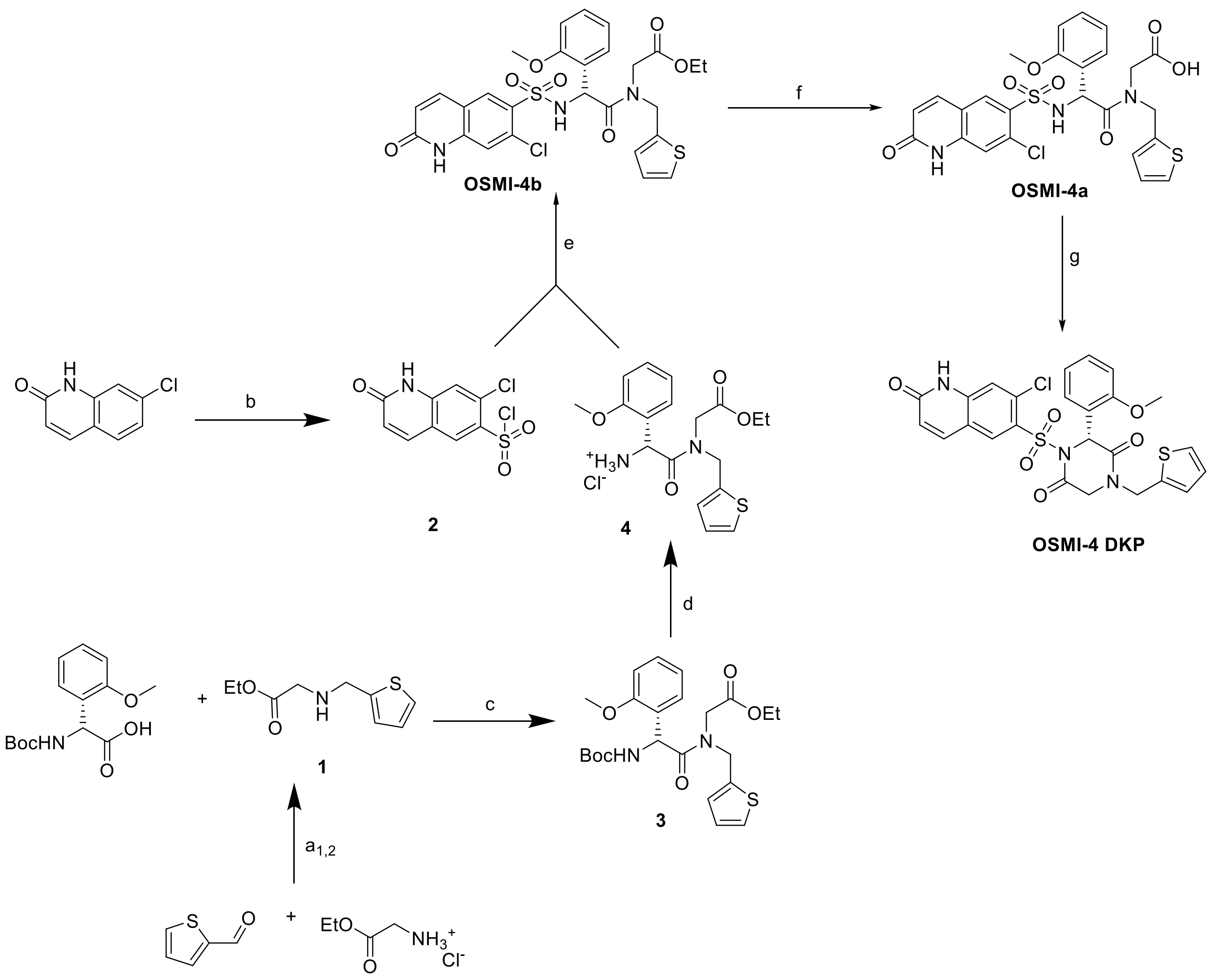

Fig. S1 Synthesis of OSMI-4b and derivatives OSMI-4a and OSMI-4 DKP. a1) Et_3N, EtOH, rt, 90min; a2) $NaBH_4$, overnight 58%; b) HSO_3Cl, 148°C, 3h, 27 %; c) HATU, DIPEA, DMF, rt, 77%; d) HCl, THF, 2h, rt; e) DIPEA,DMF, rt, 98%; f) LiOH, THF, 4°C, 2h, 79%; g) HATU, DIPEA, DMF, 0°C, rt, 24%.

Methyl (thiophen-2-ylmethyl)glycinate (**1**);

Glycine ethyl ester hydrochloride (3,49 g, 25 mmol) was dissolved in absolute EtOH (100 mL) and Et3N (3,5 mL, 25 mmol) was added via syringe, followed by thiophene 2-carboxaldehyde (2,28 mL, 25 mmol). The reaction was stirred for 2 h at 40 °C, then allowed to cool down to 0 °C using an ice bath. Sodium borohydride (1,89 g, 50 mmol) was slowly added to the mixture, then the ice bath was removed and the reaction was stirred at room temperature overnight. A black precipitate was filtered off and the filtrate was concentrated under reduced pressure. The residue was partitioned between EtOAc (50 mL) and H2O (50 mL). The organic layer was washed with H2O (2 × 40 mL) and brine (40 mL), then dried with Na2SO4. EtOAc was removed in vacuo and the residue was purified using flash silica chromatography, eluting with 5-50% EtOAc/Hexanes to yield 2,88 g (58%) of **1** as a yellow oil. 1H NMR (400 MHz, CDCl3) δ 7.23 (dd, J = 4.8, 1.5 Hz, 1H), 6.97 – 6.92 (m, 2H), 4.19 (q, J = 7.1 Hz, 2H), 4.03 (d, J = 0.7 Hz, 2H), 3.44 (s, 2H), 1.28 (t, J = 7.1 Hz, 3H).

7-chloro-2-oxo-1,2-dihydroquinoline-6-sulfonyl chloride (**2**);

A round bottom flask was charged with 7-chloroquinolin-2(1*H*)-one (1,72 g, 4.2 mmol), capped with a septum cap and filled with argon. Chlorosulphonic acid was added via syringe and the suspension was heated at 148 °C for 3 h. The reaction mixture was allowed to cool down to room temperature, and was then poured into crushed ice. The precipitate was collected by filtration, washed with cold water, and triturated with ethyl acetate. It was then dried under reduced pressure to afford 7-chloro-2-oxo-1,2-dihydroquinoline-6-sulfonyl chloride as a light brown solid. (0,710 g, 27 % yield). ^{1}H NMR (400 MHz, DMSO-d_6) δ 11.82 (s, 1H), 8.16 (s, 1H), 7.98 (d, *J* = 9.5 Hz, 1H), 7.28 (s, 1H), 6.49 (d, *J* = 9.5 Hz, 1H).

Ethyl (*R*)-*N*-(2-((tert-butoxycarbonyl)amino)-2-(2-methoxyphenyl)acetyl)-*N*-(thiophen-2-ylmethyl)glycinate (**3**);

(*R*)-2-((tertbutoxycarbonyl)amino)-2-(2-methoxyphenyl)acetic acid (450 mg, 1,71 mmol) and **1** (341 mg, 1,71 mmol) were cooled to 0°C under argon atmosphere for 10 minutes. Dimethylformamide (DMF) was added via syringe (12 mL), followed by 1-[Bis(dimethylamino)methylene]-1H-1,2,3-triazolo[4,5-b]pyridinium 3-oxide hexafluorophosphate (HATU) (715 mg, 1,88 mmol) by briefly removing the septum cap. Diisopropylethylamine (328 µL, 1,88 mmol) was added via syringe, and the reaction was stirred at rt overnight. The reaction mixture was partitioned between EtOAc (10 mL) and H_2O (10 mL). The H_2O layer was extracted with fresh EtOAc (2 × 10 mL). The organic layers were combined, washed with brine (10 mL), dried over Na_2SO_4, and concentrated under reduced pressure. The residue was purified by flash chromatography, eluting with 30-50% EtOAc/Hexanes to yield 677 mg (77%) of **3** as a light yellow oil (mixture of rotamers). ^{1}H NMR (400 MHz, DMSO-d_6) δ 7.50 – 7.39 (m, 1H), 7.37 – 7.23 (m, 2H), 7.06 – 6.85 (m, 4H), 5.85 (dd, *J* = 119.7, 8.9 Hz, 1H), 4.73 – 4.53 (m, 2H), 4.17 – 3.98 (m, 3H), 3.76 (d, *J* = 6.2 Hz, 3H), 1.36 (s, 9H), 1.16 – 1.09 (m, 3H).

Ethyl (*R*)-*N*-(2-((7-chloro-2-oxo-1,2-dihydroquinoline)-6-sulfonamido)-2-(2-methoxyphenyl)acetyl)-*N*-(thiophen-2-ylmethyl)glycinate (**4** and **OSMI-4b**);

5 mL of HCl (4M solution in Dioxane) was added to **3** (160 mg, 0.35 mmol) at 0°C under argon atmosphere. The reaction was stirred at room temperature for 1 hour, then it was concentrated under reduced pressure to yield compound **4** as a clear oil.

Compound **2** (0.36 mmol, 100 mg) was dissolved in 2.5 mL of dry dimethylformamide under argon atmosphere. A solution of compound **4** (0.24 mmol, 87 mg) in dry dichloromethane (0.8 mL) was added by syringe, followed by DIPEA (150 μL). The reaction was stirred at room temperature overnight and then concentrated under vacuum. The residue was partitioned between ethyl acetate and water. The water phase was extracted with fresh ethyl acetate, then the organic layers were combined, washed with brine, dried over Na_2SO_4 and concentrated under reduced pressure. The resulting oil was purified by flash chromatography, eluting with 25-60% EtOAc/Toluene plus 5% ethanol to yield 140 mg (98%) of OSMI-4b as a white solid (1:1 mixture of rotamers). HPLC 99% pure.^{1}H NMR (400 MHz, $CDCl_3$) δ 12.42 (s, 1H), 7.98 (d, J = 1.4 Hz, 1H), 7.58 (dd, J = 9.5, 3.3 Hz, 1H), 7.34 – 7.09 (m, 5H), 6.94 – 6.78 (m, 2H), 6.75 – 6.61 (m, 2H), 6.08 (d, J = 145.0 Hz, 1H), 5.02 – 4.51 (m, 2H), 4.18 – 4.08 (m, 1H), 4.04 – 3.95 (m, 1H), 3.93 – 3.85 (m, 2H), 3.79 (d, J = 8.43 Hz, 3H), 1.17 (dt, J = 14.7, 7.1 Hz, 3H).
^{13}C NMR (400 MHz, $CDCl_3$) δ 168.26, 167.79, 163.85, 155.81, 155.61, 140.60, 140.08, 137.85, 137.20, 133.54, 133.47, 130.69, 130.66, 130.51, 130.30, 128.97, 128.79, 127.54, 127.26, 126.96, 126.61, 126.36, 126.29, 122.58, 121.47, 121,33, 117.99, 117.02, 117.00, 111.05, 110.71, 61.60, 61.35, 55.79, 55.62, 46.91, 46.75, 46.13, 45.41, 29.73, 14.15, 14.00.

(*R*)-*N*-(2-((7-chloro-2-oxo-1,2-dihydroquinoline)-6-sulfonamido)-2-(2-methoxyphenyl)acetyl)-*N*-(thiophen-2-ylmethyl)glycine (**OSMI-4a**);
A solution of **OSMI-4b** (0,166 mmol, 100 mg) in tetrahydrofuran (2 mL) was cooled to 4°C before adding LiOH 1 M (1,66 mmol, 1,7 mL). The reaction was stirred for 3 hours at 4 °C then THF was removed under reduced pressure and the residue was partitioned between water and ethyl acetate. The aqueous layer was acidified with fuming HCl and extracted with fresh ethyl acetate. The organic phases were combined, dried over Na_2SO_4 and concentrated. The residue was then dissolved in a EtOAc/Hexane mixture to recrystallise the desired product **OSMI-4a** (45 mg). The aqueous layer was concentrated in vacuo, resuspended in acetone, filtered and concentrated again, to yield 30 mg of product. (79%). HPLC 97% pure.

(*R*)-4-((7-chloro-2-hydroxyquinolin-6-yl)sulfonyl)-3-(2-methoxyphenyl)-1-(thiophen-2-ylmethyl)piperazine-2,5-dione (**OSMI-4 DKP**);
OSMI-4a (0,08 mmol, 44 mg) and 1-[Bis(dimethylamino)methylene]-1H-1,2,3-triazolo[4,5-b]pyridinium 3-oxide hexafluorophosphate (HATU) (0,09 mmol, 32 mg) were dissolved in DMF (2 mL) under argon atmosphere at 0 °C. DIPEA (0,09 mmol, 11 μL) was added to the mixture via syringe and the reaction was stirred at room temperature overnight. DMF was removed in vacuo and the residue was partitioned between ethyl acetate and water. The organic layer was then washed with brine and dried over Na_2SO_4. The crude product was purified by flash chromatography eluting with 5% MeOH/DCM, to yield **OSMI-4 DKP** as a white solid (10 mg, 24%). HPLC 95% pure. ^{1}H NMR (400 MHz, $CDCl_3$) δ 8.27 (s, 1H), 7.72 (d, *J* = 9.6 Hz, 1H), 7.51 – 7.46 (m, 1H), 7.35 – 7.29 (m, 2H), 7.20 (dd, *J* = 5.1, 1.2 Hz, 1H), 6.97 (t, *J* = 7.5 Hz, 1H), 6.92 (d, *J* = 3.3 Hz, 1H), 6.88 (dd, *J* = 5.1, 3.4 Hz, 1H), 6.83 (d, *J* = 8.3 Hz, 1H), 6.62 (d, *J* = 9.6 Hz, 1H), 5.94 (s, 1H), 4.95 (d, *J* = 15.1 Hz, 1H), 4.39 (d, *J* = 15.1 Hz, 1H), 4.05 (d, *J* = 18.2 Hz, 1H), 3.81 (d, *J* = 18.1 Hz, 1H), 3.47 (s, 3H). ^{13}C NMR (600 MHz, $CDCl_3$) δ 163.86, 163.83, 163.28, 156.53, 142.49, 139.99, 136.28, 134.22, 133.02, 132.79, 131.12, 129.19, 128.07, 126.79, 126.68, 123.50, 123.44, 121.12, 118.18, 117.64, 111.12, 61.45, 55.06, 49.85, 44.17.

Cellular permeability

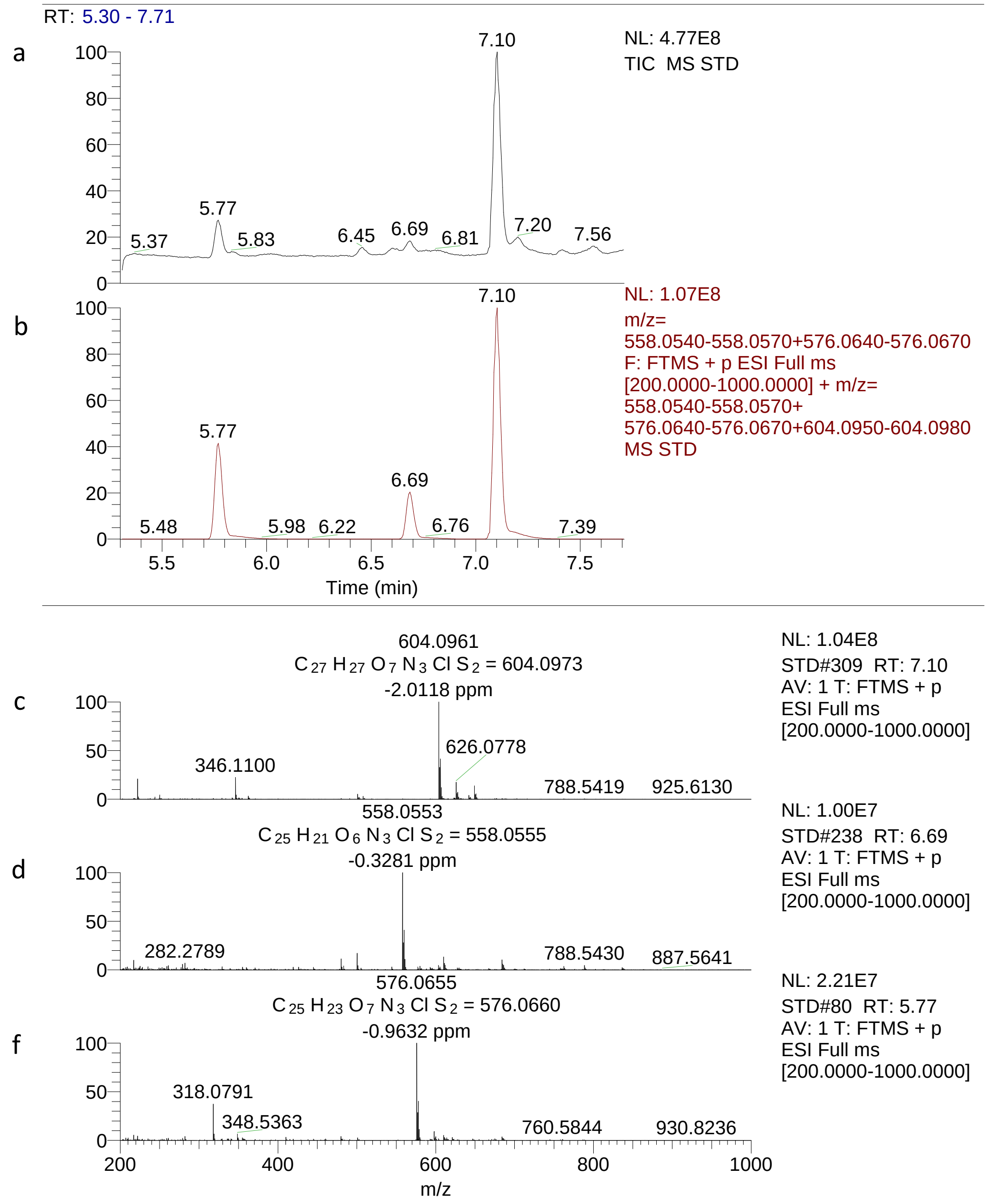

Fig. S2 Total ion count (TIC) (a) and extracted ion chromatogram (EIC) (b) of a mixture of ester OSMI-4b (RT = 7.10 min), acid OSMI-4a (RT = 5.77 min) and diketopiperazine derivative OSMI-4 DKP (RT = 6.69 min). Mass spectra recorded at 7.10 min (c), 6.69 (d) and 5.77 min (f) comfirmed retention time of each compound. Mixture was prepared by dissoving compounds in 20% methanol, concentration of each compound was 1 mg/L.

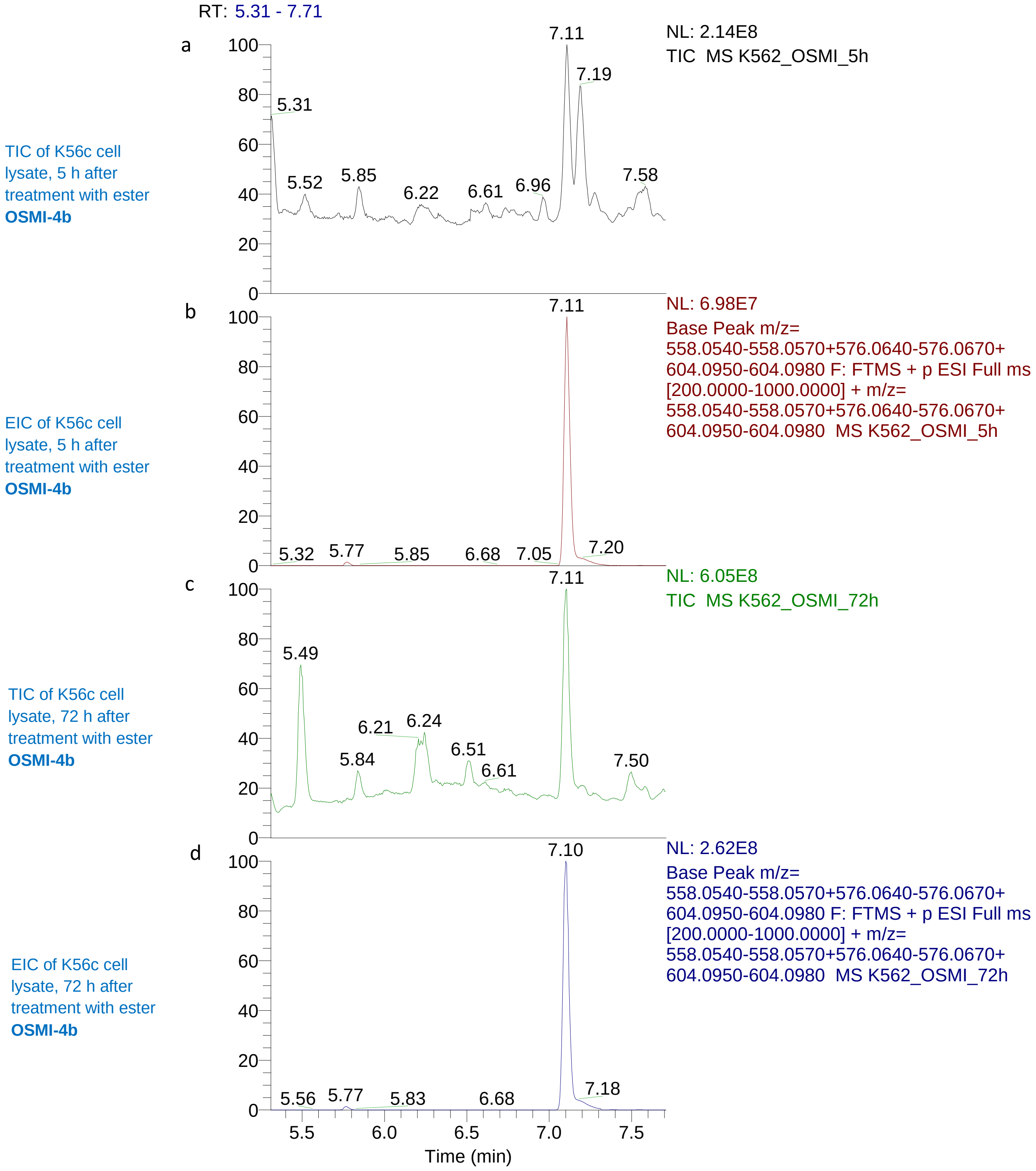

Fig. S3 TIC and EIC of cell lysates of K562 cell line after treating cells with ester OSMI-4b for 5 h (a and b) or 72 h (c and d). Samples were prepared in 20% methanol.

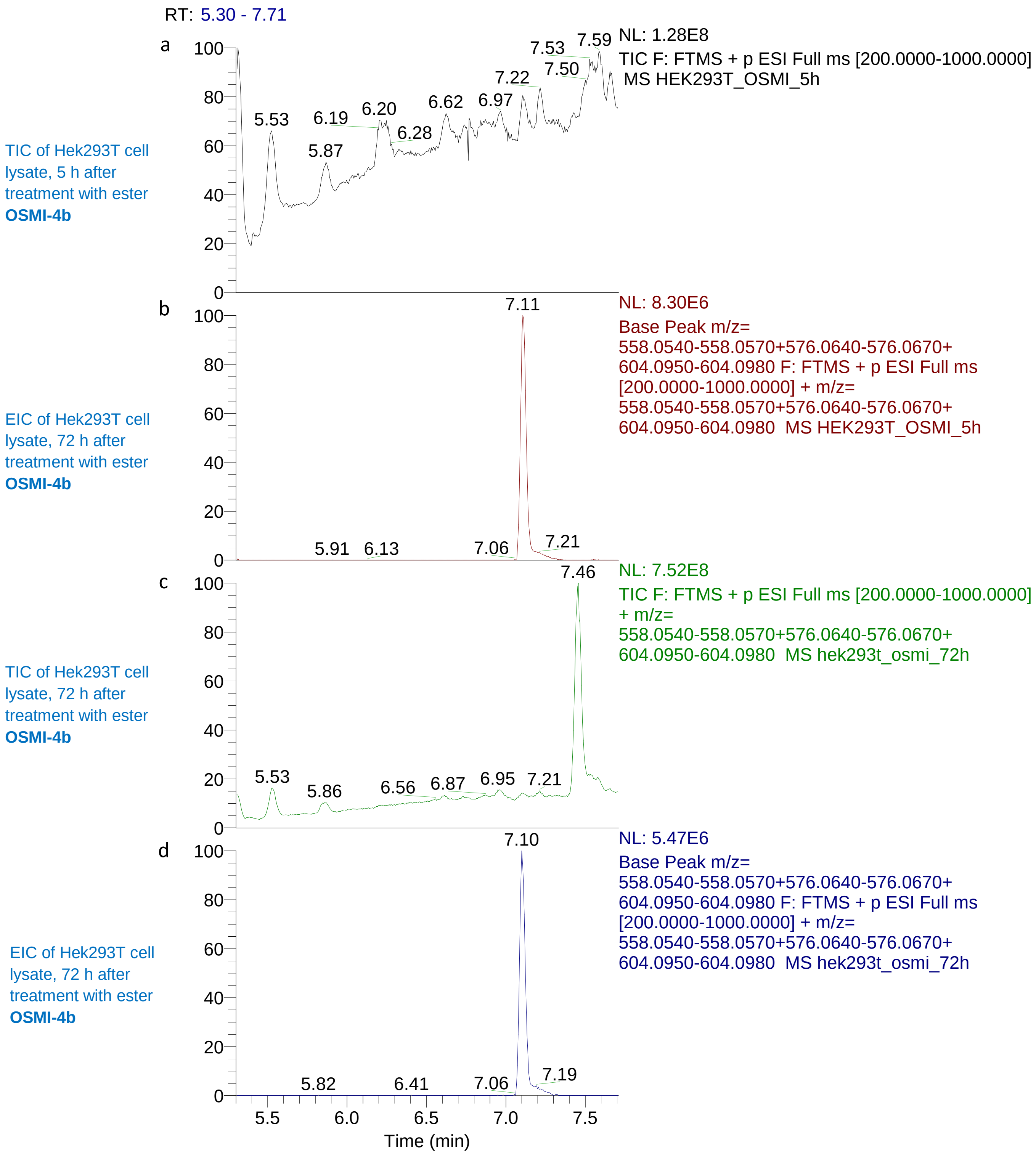

Fig. S4 TIC and EIC of cell lysates of Hek293T cell line after treating cells with ester OSMI-4b for 5 h (a and b) or 72 h (c and d). Samples were prepared in 20% methanol.

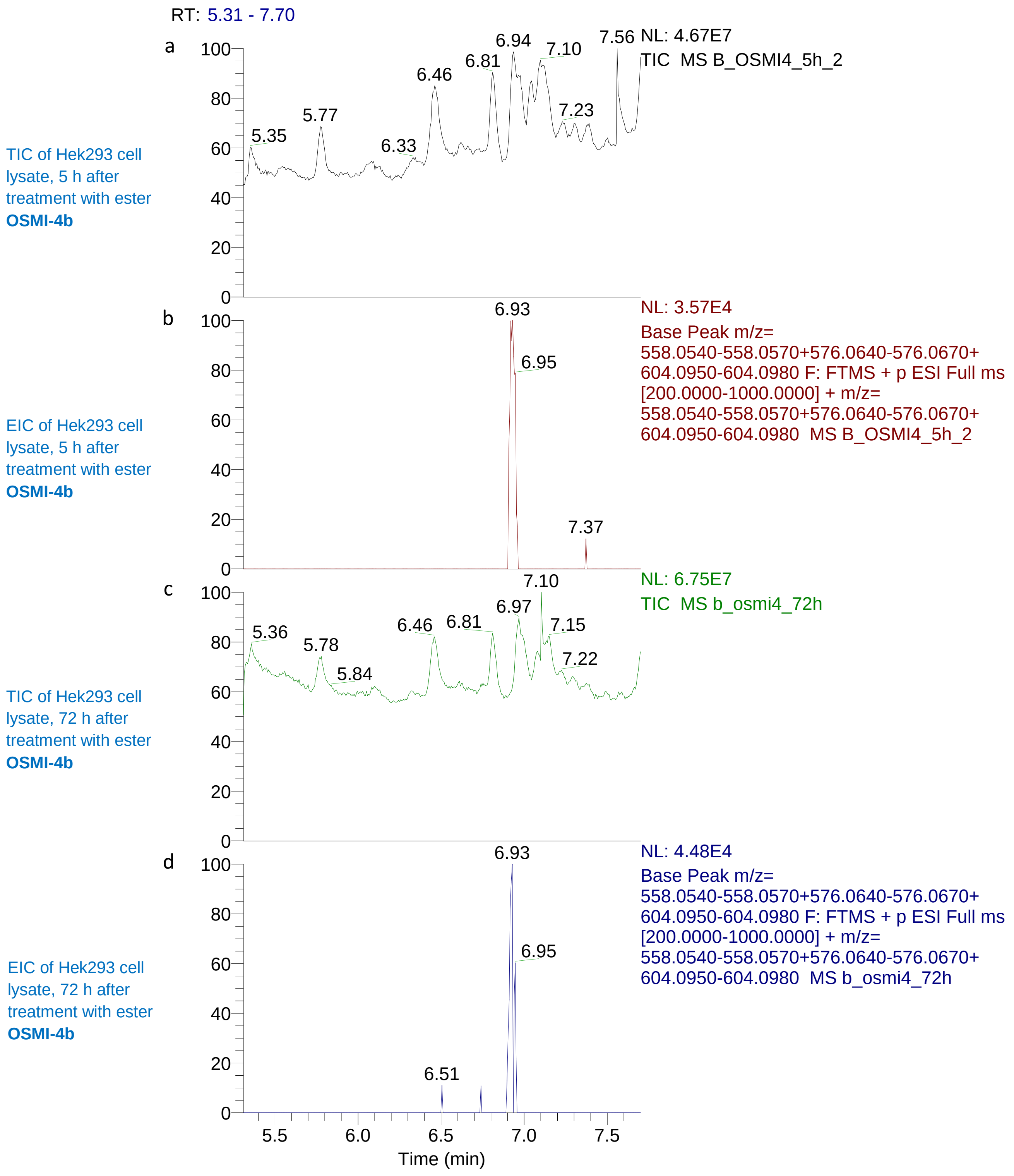

Fig. S5 TIC and EIC of cell lysates of Hek293 cell line after treating cells with ester OSMI-4b for 5 h (a and b) or 72 h (c and d). Samples were prepared in 50% acetonitrile (standard in figure S6).

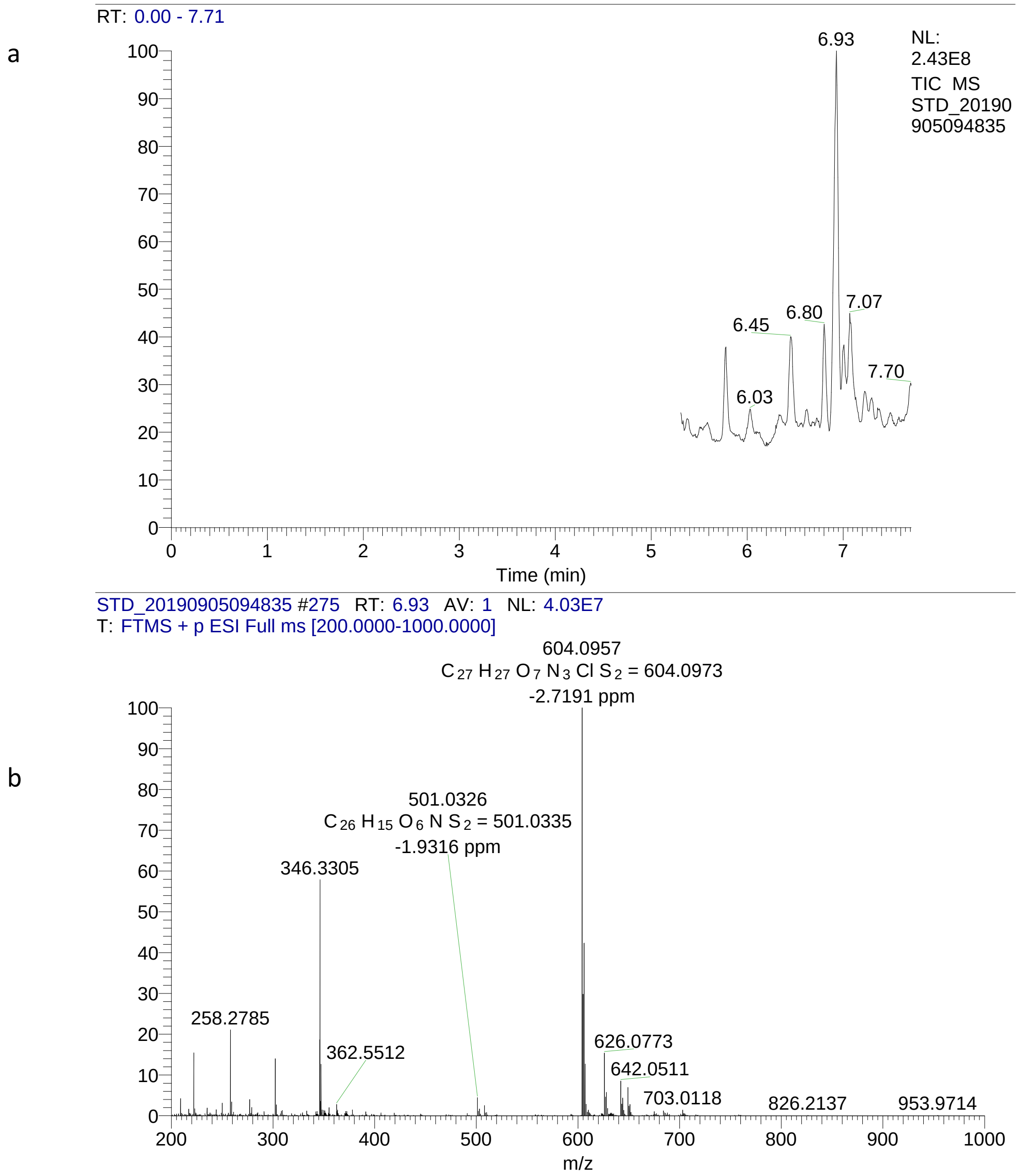

Fig. S6 TIC (a) and EIC (b) of ester OSMI-4b (RT = 6.93 min). Retention time was confirmed with mass spectrum recorded at 6.93 min. Sample was prepared in 50% acetonitrile, concentration of compound OSMI-4b was 1 mg/L.

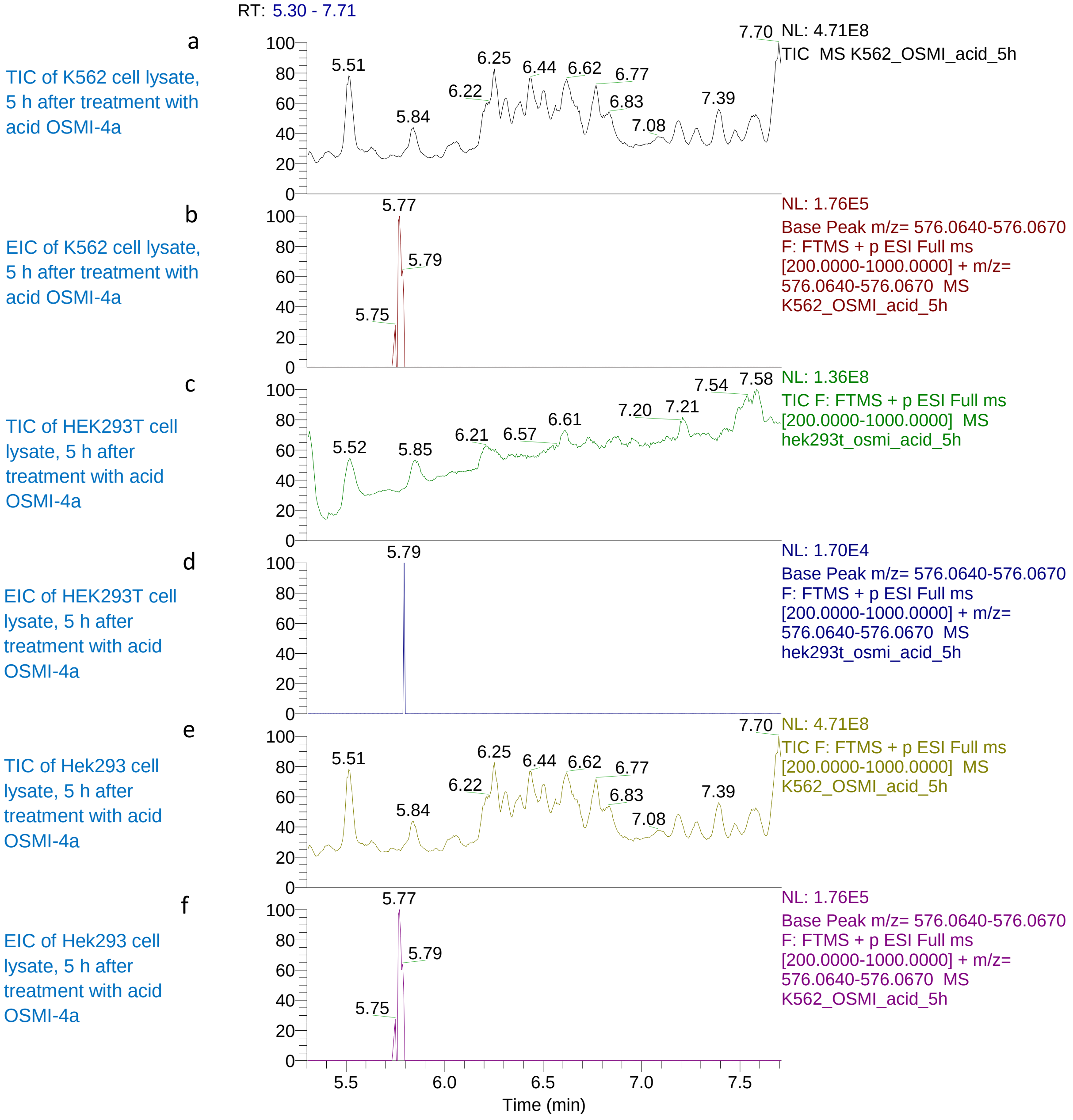

Fig. S7 TIC and EIC of cell lysates of K562 (a and b), HEK293T (c and d) and HEK293 (e and f) cell lines after treating cells with acid OSMI-4a for 5 h. Samples were prepared in 20% methanol. Peaks corresponding to OSMI-4a in extracted ion chromatograms b, d and f are near the limit of detection.

IC₅₀ measurement with fluorescent activity assay

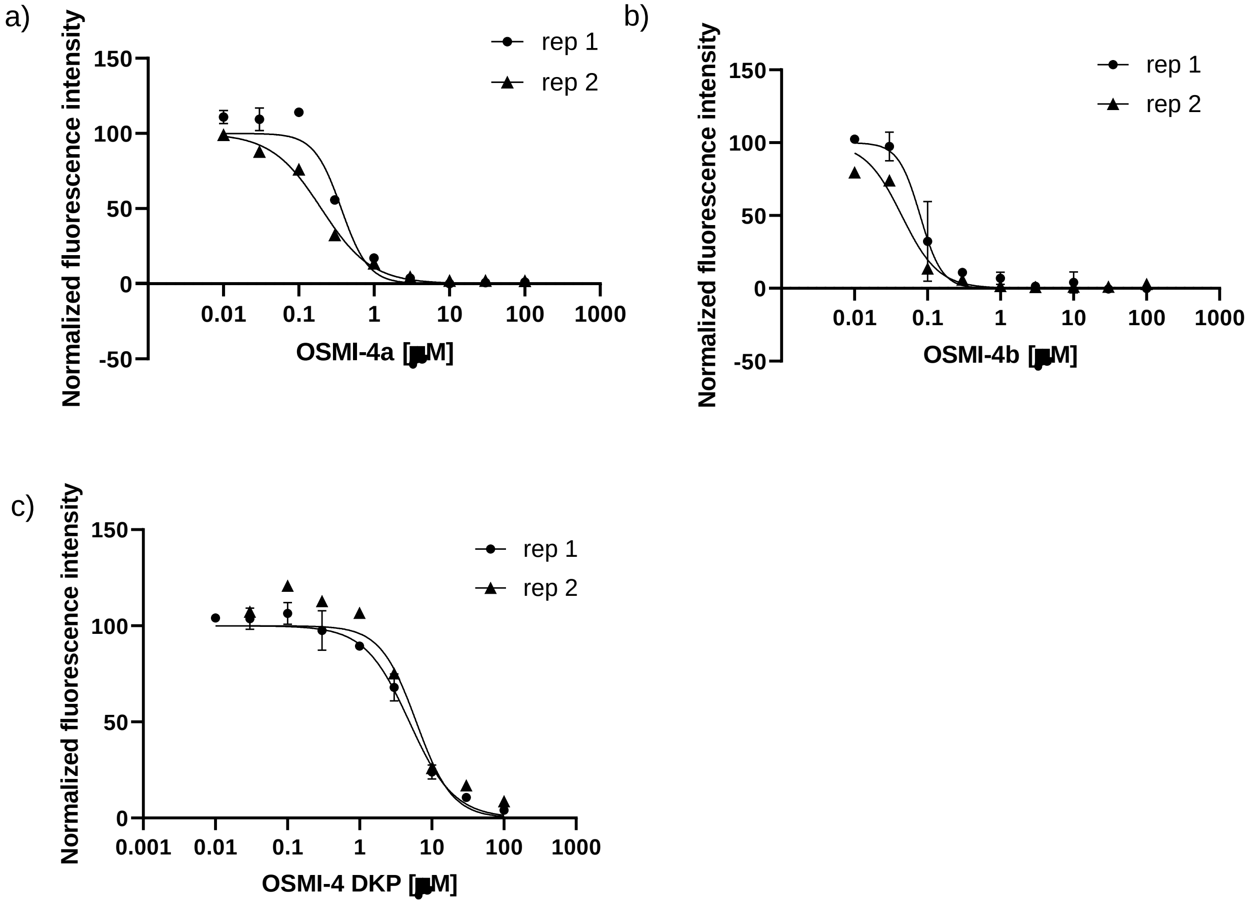

Fig. S8 IC_{50} curves of a) OSMI-4a, b) OSMI-4b, c) OSMI-4 DKP measured in two independent experiments. Normalized fluorescence intensity values are expressed as a percentage of the DMSO control.

IC₅₀ measurement with UDP-Glo assay

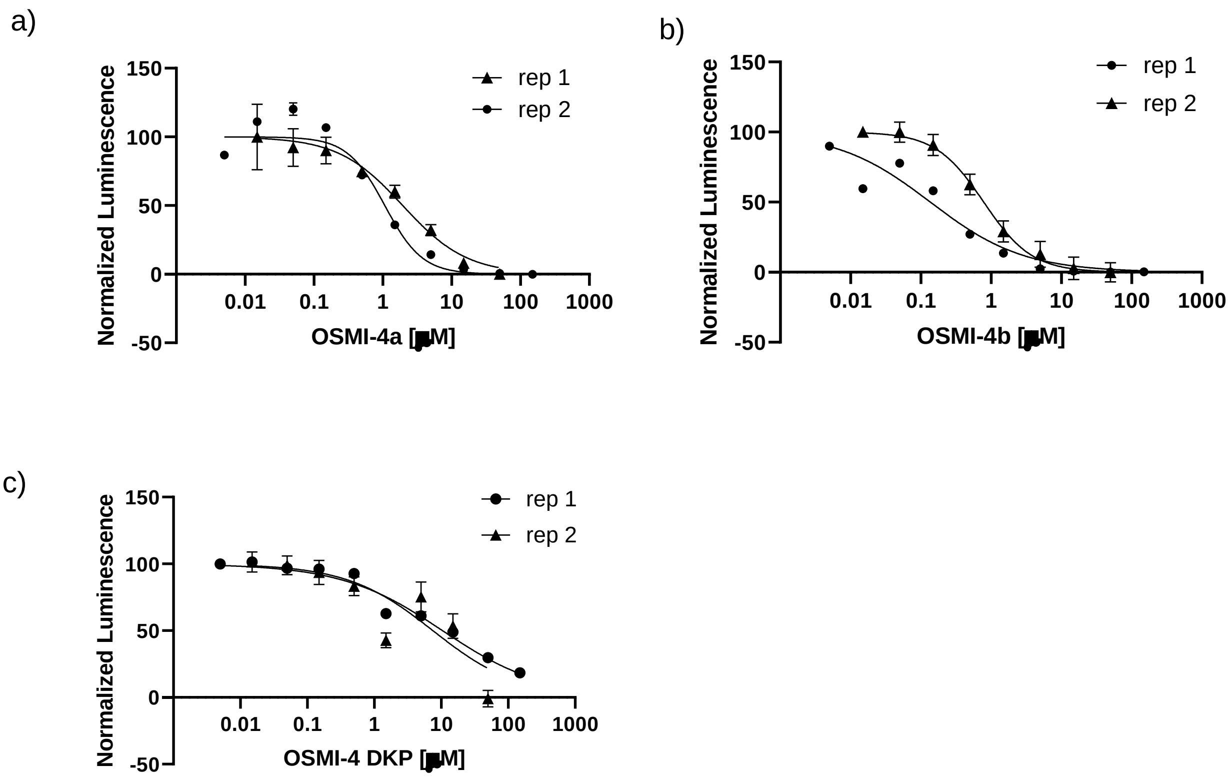

Fig. S9 IC_{50} curves of a) OSMI-4a, b) OSMI-4b, c) OSMI-4 DKP measured in two independent experiments. Normalized luminescence values are expressed as a percentage of the DMSO control.

.
